# Immune Checkpoints, Inhibitors and Radionuclides in Prostate Cancer: Promising Combinatorial Therapy Approach

**DOI:** 10.3390/ijms22084109

**Published:** 2021-04-15

**Authors:** Mankgopo M. Kgatle, Tebatso M. G. Boshomane, Ismaheel O. Lawal, Kgomotso M. G. Mokoala, Neo P. Mokgoro, Nico Lourens, Kalevo Kairemo, Jan Rijn Zeevaart, Mariza Vorster, Mike M. Sathekge

**Affiliations:** 1 Department of Nuclear Medicine, University of Pretoria & Steve Biko Academic Hospital, Pretoria 0001, South Africa; gboshomane@gmail.com (T.M.G.B.); ismaheellawal@gmail.com (I.O.L.); kmgmokoala@gmail.com (K.M.G.M.); mokoneo@yahoo.com (N.P.M.); marizavorster@gmail.com (M.V.); 2 Nuclear Medicine Research Infrastructure (NuMeRI), Steve Biko Academic Hospital, Pretoria 0001, South Africa; janrijn.zeevaart@necsa.co.za; 3 Department of Nuclear Medicine, Steve Biko Academic Hospital, Pretoria 0001, South Africa; 4 Department of Urology, University of Pretoria & Steve Biko Academic Hospital, Pretoria 0001, South Africa; nico.lourens@up.ac.za; 5 Departments of Molecular Radiotherapy & Nuclear Medicine, Docrates Cancer Center, 00180 Helsinki, Finland; kalevo.kairemo@gmail.com; 6 Department of Nuclear Medicine, The University of Texas MD Anderson Cancer Center, Houston, TX 77030, USA; 7 Radiochemistry, South African Nuclear Energy Corporation SOC (Necsa), Pelindaba 0001, South Africa

**Keywords:** immune checkpoints, immune checkpoints inhibitors, immunotherapy, metastatic hormone resistance prostate cancer, metastatic hormone sensitive prostate cancer, prostate cancer, peptide receptor ligand therapy, radionuclides, prostate specific membrane antigen

## Abstract

Emerging research demonstrates that co-inhibitory immune checkpoints (ICs) remain the most promising immunotherapy targets in various malignancies. Nonetheless, ICIs have offered insignificant clinical benefits in the treatment of advanced prostate cancer (PCa) especially when they are used as monotherapies. Current existing PCa treatment initially offers an improved clinical outcome and overall survival (OS), however, after a while the treatment becomes resistant leading to aggressive and uncontrolled disease associated with increased mortality and morbidity. Concurrent combination of the ICIs with radionuclides therapy that has rapidly emerged as safe and effective targeted approach for treating PCa patients may shift the paradigm of PCa treatment. Here, we provide an overview of the contextual contribution of old and new emerging inhibitory ICs in PCa, preclinical and clinical studies supporting the use of these ICs in treating PCa patients. Furthermore, we will also describe the potential of using a combinatory approach of ICIs and radionuclides therapy in treating PCa patients to enhance efficacy, durable cancer control and OS. The inhibitory ICs considered in this review are cytotoxic T-lymphocyte antigen 4 (CTLA4), programmed cell death 1 (PD1), V-domain immunoglobulin suppressor of T cell activation (VISTA), indoleamine 2,3-dioxygenase (IDO), T cell Immunoglobulin Domain and Mucin Domain 3 (TIM-3), lymphocyte-activation gene 3 (LAG-3), T cell immunoreceptor with Ig and ITIM domains (TIGIT), B7 homolog 3 (B7-H3) and B7-H4.

## 1. Introduction

PCa PCa is the second most frequently diagnosed malignancy, and the sixth leading cause of cancer-related deaths in older men worldwide. In 2018, the incidence of PCa was approximately 1.3 million with a mortality rate of 360,000 [1]. There appears to be a positive correlation in the incidence, prevalence and increasing age of PCa; although the diagnosis of PCa remains undiagnosed in some men [2]. This may be owing to the asymptomatic PCa cases, Gleason score, screening tools, limited healthcare access and social awareness aspects in men especially in the rural settings. Additionally, routine screening procedures for PCa include digital rectal examination (DRE) for assessment of the prostate gland and use of serum prostate specific antigen (sPSA) [3]. However, these techniques are not perfect, i.e., a DRE is operator dependent and sPSA is not specific for PCa. sPSA can also be elevated in several benign and non-benign conditions (e.g., infections such as urinary tract infections, prostatitis and benign prostatic hypertrophy) which form part of PCa risk factors [4,5]. Other factors such as black ethnicity, PCa family history, having many children and diet have shown to have a positive correlation with increased risk of PCa development, although more studies are needed to determine the accuracy of some of these risk factors [3,5,6].

**Figure 1:**
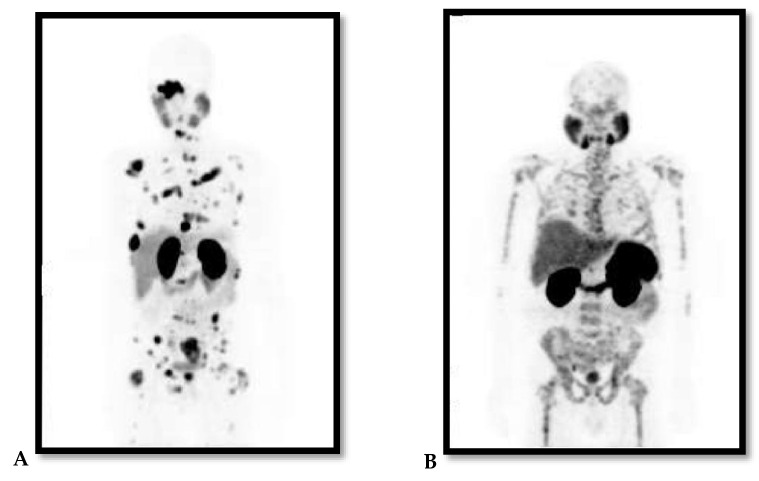
(**A** and **B** Maximum Projection images): ^68^Ga-PSMA PET images of two patients with advanced/metastatic prostate cancer to the skeleton. Note the difference in pattern of bone involvement and both are diffused skeletal demonstrating lytic and sclerotic. (**A**) demonstrates focal pattern but widespread lesions; (**B**) demonstrates lesions that are widespread in pattern interrupted with areas in the bone of no uptake within the affected bone.

PCa is a notoriously heterogeneous cancer with 60–90% of the patients having multiple distinct cancer foci within the prostate gland at time of diagnosis [7]. The heterogeneity of PCa is also observed through its metastatic predominance for the skeleton with high risk of biochemical recurrence and persistence following treatment, and this correlates with increased morbidity and mortality. This has been illustrated in Figure 1 with ^68^Gallium(^68^Ga)-prostate-specific membrane antigen (PSMA)-Positron Emission Tomography (PET) images of two patients with metastatic PCa to the skeleton, which vary in the pattern of bone involvement and histological features. This probes for personalised and targeted therapeutic approach. Most PCa diagnoses and treatments are purely reliant on increased levels of sPSA and androgen hormone (AH), which is highly detectable in localised PCa and certain cancer cells. This makes advanced or aggressive PCa such as metastatic hormone-refractory PCa (mHRPC) with suppressed levels of PSA or AH difficult to diagnose and treat [8]. When diagnosed early especially at the local stage with well-differentiated non-metastatic disease, the 5-year relative survival rate of PCa is >95% as observed in more than 3000 patients with a new diagnosis recorded in the Norwegian PCa Registry in 2004–2005 [9]. PCa is classified into low, intermediate and high-risk groups, and this based on TNM stage that describes the amount and spread of cancer in patient’s body, Gleason score and level of PCa marker called sPSA [8,10].

**Figure 2:**
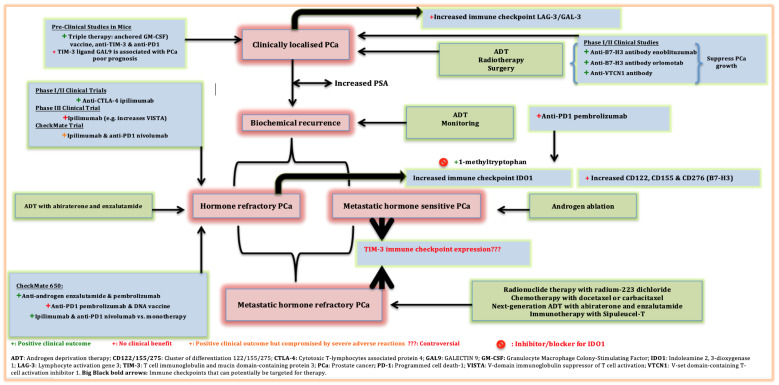
Clinical disease progression, pathophysiology and current therapeutic interventions of prostate cancer. Most PCa patients usually progresses slowly to mHRPC or mHSPC. PCa treatment varies according to stages and pathophysiology of the disease, and these currently include chemotherapy, radiotherapy, surgery and ADT. Various preclinical and clinical trials have been tested for immunotherapy approaches targeting various immune checkpoints although these have demonstrated minimal clinical benefit in PCa especially when used as monotherapies. **Bold arrows** represent immune checkpoints that can potentially be targeted for therapy. Color codes: pink boxes—clinical disease progression; green boxes—established therapy strategies; blue boxes—novel therapy option.

PCa patients are usually at risk of developing further biochemical recurrence (BR) and disease progression following radical prostatectomy (RPT) and salvage radiotherapy (SRT). The recurrent and progressive metastasis are often located outside the prostate fossa with a low-volume disease burden, suggesting metastasis-directed therapy [7]. The PSMA-PET imaging is currently revolutionising the way we image and treat PCa and bears the promise of delivering individually tailored targeted radiotherapy. For instance, advanced PCa or mHRPC characterised by distant organ metastases may be controlled with a combination of radionuclide therapy with radium-223 (223Ra) dichloride, chemotherapy with docetaxel/cabazitaxel (ALSYMPCA trial) and surgery to maximise local control. These therapies are associated with survival benefits as illustrated in Figure 2 and Table 1 [11,12,13]. Androgen deprivation therapy (ADT) is categorised in first generation and next/new generation ADT. First generation ADT is useful for metastatic hormone-sensitive PCa (mHSPC). Next generation ADT is the cornerstone treatment for HRPC that involves cancer cells that are no longer responsive to first generation ADT. However, next/new generation ADT can be applied in the setting of HSPC and HRPC, and its benefit has been shown by its ability to induce immune microenvironment remodelling that positively correlates with recurrence free-survival (RFS) and immune infiltration [14]. Nonetheless, ADT still provides minimal substantial clinical benefit for all PCa patients because it only targets the AR pathway [15].

## 2. Recap the Role of Immune System in PCa and Advanced Stages

Chronic inflammation and immunosuppression are the hallmarks of many cancers, and are also implicated in PCa [46,47,48]. Recent studies demonstrate that IC suppression remains significantly relevant in PCa and may be efficacious in PCa patients with advanced malignancy when used in combination with other treatment modalities [47,49,50]. the ICs are immunoregulators that control the activities of T cell response by activating co-stimulatory and inhibitory immune signals. The T cells, B cells, natural killer (NK) cells, dendritic cells (DCs) and macrophages are types of immune cells, which are a network of specialised organs, tissues, cells and signalling molecules that synergise as powerful weapons to fight pathogens and cancers [47]. T cells form one of the major components of adaptive immunity that elicit responses by activating and attacking damaged cells including dendritic cells, B-cells and macrophages that have digested foreign antigens. ICs on the T cell surfaces serve as gatekeepers that control the activities of the T cell response. In normal circumstances, ICs maintain the inactive status of T cells (as naïve T cells) to prevent them from attacking and damaging the body’s own tissues or cells until they encounter specific foreign antigens [51,52,53]. Classified as either self or non-self, antigens enable the immune system to distinguish between normal interactions and antigen encounter with the foreign threat. Neoantigens are types of tumour antigen derived from mutations and tumour cells/DNA and are recognisable as non-self by the immune system. In both innate and adaptive immune responses, immune cells recognise and eliminate tumour cells in 3 principal stages [54,55]. The first stage is presentation, and in this stage the innate immune responses (neutrophils, basophils, eosinophils, and macrophages) rapidly identifies and attacks tumour cells. The resulting tumour cell death release tumour antigens, which can activate the cytotoxic T cells of the adaptive immune system. The second stage is infiltration, and it involves recruitment of immune cells by tumour antigens and other factors to the tumour site, where they invade and attacks the tumour cells. Elimination is the last stage, and here activated cytotoxic T cells recognise the tumour cells as the source of antigens and target them for elimination [54,55,56].

**Figure 3:**
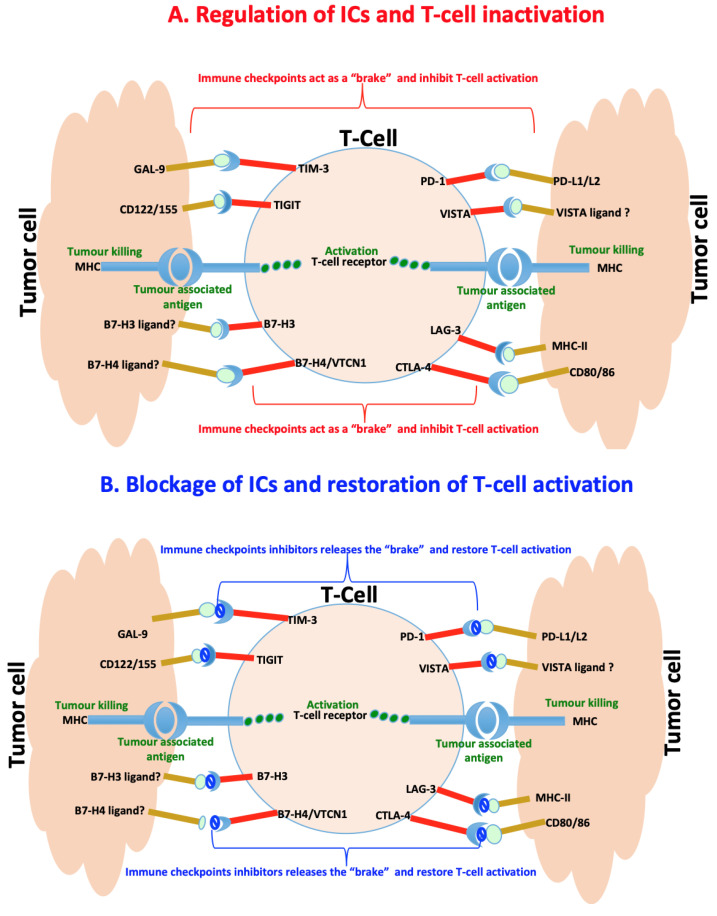
Immunoregulation of checkpoints in prostate cancer. (**A**) Immune checkpoints are usually hijacked during cancer and act as a “brake” upon binding to their ligands to slow down or inhibit cancer targeting T-cells and killing of tumor cells. (**B**) Counteracting the interaction of the immune checkpoints by their specific inhibitors release the “brake” and restores the T-cells functioning leading to killing of tumour cells.

The immunosuppression activity of the immune cells such as Tregs and myeloid-derived suppressor cells (MDSC) is regulated by activating and inhibitory pathways. Activating pathways trigger an immune response. Inhibitory pathways such as IC pathways provide a natural counterbalance to immune activation by serving as “brakes” of the immune system that tumours usually hijack in order to shut down immune responses and protection [51,52,53,57]. This balance between inhibitory and activating pathways normally enables the immune system to attack tumour cells while sparing healthy normal cells. However, tumour cells may modulate these pathways in order to escape the detection and destruction by these immune effector cells [58,59,60]. Blockage of these pathways, especially inhibitory that tend to be implicated in various types of cancer, has shown potential therapeutic benefits by producing antitumour effects and long-term survival benefits in a broad spectrum of cancers [61,62]. This inspired the development of ICIs or blockades that led to the revolutionary treatment of many cancer types and earned both James P Allison and Tasuku Honjo a 2018 Nobel Prize in Physiology or Medicine. Patients with melanoma, bladder and kidney cancers that exhibit mismatch repair deficiency (dMMR), cyclin dependent kinase 12 (CDK12) loss and high tumour mutational burden characterised by good T cell infiltration tend to respond well to ICIs as compared to patients with PCa [63,64]. PCa is generally immunologically “cold” associated with low tumour mutational burden around tumour microenvironment and enriched with poor T cell infiltration and myeloid cells that are immunosuppressive [65,66]. However, the use of double- instead of single-agent monotherapy of ICIs or combination of single-agent monotherapy ICIs with other PCa treatment including the PCa vaccine sipuleucel-T tend to give better clinical outcomes [63,67,68]. It is, therefore, important to understand the role of inhibitory pathways in PCa to open new avenues for the development of dual combination therapeutic approaches that will favour all patients and offer a better clinical outcome. Here, we focus primarily on the recent progress in understanding inhibitory IC pathways, their inhibitors and roles in PCa treatment. These will include but is not limited to CTLA4, PD1, VISTA, IDO1, TIM3, LAG3, TIGIT, B7-H3 and B7-H4 and these are illustrated in Figure 3.

## 3. The Immunoregulation and Inhibition of Immune Checkpoints in PCa

### 3.1. Cytotoxic T-lymphocyte Antigen 4

CTLA4 CTLA4 and PD1 pathways have been heavily studied, and ICIs that are under clinical studies today target these pathways or their ligands to restore antitumour responses. CTLA4 is constitutively expressed on regulatory T cells (Tregs), normal and malignant non-T cells leading to an integration network complex of positive and negative co-stimulatory signals that are required for T cell modulation. Two positive signals are required for T cell activation, and these include antigen presentation and CD28 to initiate the immune response [69]. CTLA4 and CD28 are homologous receptors both expressed by CD4+ and CD8+ T-cells which mediate opposing functions in T cell activation. They share APC expressed CD80 (B7-1) and CD86 (B7-2) as their natural ligands. Transient expression of CTLA4 occurs soon after T cell activation, resulting from ~20 times greater affinity interaction to CD80 and CD86 ligands than CD28. Thus CTLA4 outcompetes and scavenges CD80/86 away from CD28, thereby preventing CD28-mediated T cell costimulation. Subsequently, CTLA4 exerts negative inhibitory signalling to T cells by blocking CD28 co-stimulatory signal necessary for robust T cell activation and effector function. CTLA4 may also trigger trans-endocytosis and degradation of CD80 and CD86 from the cell surfaces of APC, therefore resulting in impaired costimulation via CD28-expressing T cells [70].

CTLA4 mediated T cells inhibitory signalling has been an important phenomenon implicated in various types of infections and tumours [71]. When the CTLA4 binds to its ligands, the T cells become deactivated and fail to mount the immune responses to infections and tumours. However, the blockade in CTLA4 via anti-CTLA4 ICIs is critical in disrupting the proper function of Tregs. Anti-CTLA4 binds to CTLA4 with higher affinity leading to increased accumulation, function and survival of T cells that attack tumour cells [72]. The clinical success of anti-CTLA4 ICIs was observed in advanced melanoma through the use of ipilimumab, a fully humanised antibody anti-CTLA4 monoclonal antibody (IgG) isolated from transgenic mice and produced from a hybridoma clone [73]. Ipilimumab infiltrates and represses T cell inactivation by binding to CTLA4 and preventing it from interacting with its ligands. This enables the expansion of naturally developing melanoma-specific and cytotoxic T cells that neutralise tumour cells and prevent the risk of cancer recurrence [74,75,76]. Although early phase I/II clinical trials of ipilimumab in HRPC have shown promising results with reduced cancer growth in some patients, phase III trials failed to demonstrate OS benefit [32,73,77]. Combination of ipilimumab with other therapies like docetaxel, radiotherapy, ADT, PROSTVAC, GVAX, GM-CSF resulted in PSA decline of >50% in 16% to 50% of the treated cohort, also suggesting irrelevant clinical benefit [28,29,78,79,80]. There was also no significant change in PSA doubling time when the tremelimumab was combined with ADT [81]. Poor clinical benefits of these combined therapies are likely to be attributable to the immunologically “cold” nature of tumour with relatively few tumour infiltrating T cells as mentioned earlier. However, CheckMate Trial in combination with ipilimumab and other inhibitors like nivolumab became a game changer by showing antitumour activities in both chemotherapeutic-naïve and chemotherapy-experienced HRPC patients. This was even enriched in patients with higher tumour mutational burden who benefited mostly from this treatment combination. This impressive data was compromised by observed adverse reactions (diarrhea, hypothyroidism, fatigue, skin rash, etc.) and fatalities that intercepted the use of this approach [82]. More trials with modified treatment dosage and duration approaches are ongoing to minimise these adverse reactions associated with nivolumab and ipilimumab dual-combination treatment.

### 3.2. Programmed Cell Death Protein 1

PD1 PD1 is an inhibitory receptor, an extended family of CTLA4/CD28 T cell regulators with two ligands including PD-L1 and PD-L2. PD1 is constitutively expressed by regulatory T cells, B cells, natural killer cells and certain myeloid cell populations, suggesting that its functional activities may be extended further than CTLA4. It is predominantly expressed by mature T cells in peripheral tissues and in the tumour microenvironment. Its pivotal roles involve balancing protective immunity and immunopathology, homeostasis and tolerance by modulating T cells response and possibly other immune cells through mechanisms that are still unknown [83]. PD1 knockout mouse exhibits significant altered immune cell development associated with autoimmune disease and congestive heart failure [84].

PD1 expression can limit protective immunity in responses to chronic infections and tumours [83]. Under normal physiological conditions, PD1 interacts with its ligands and recruits Src homology 2 (SH2) domain containing phosphatases 1/2 (SHP1/2) resulting in T cell immune suppression. Upon conventional T cells activation in response to chronic infections and various types of tumours, PD1 becomes upregulated and hijacked by some tumour cells to establish immune evasion. Immunohistochemistry studies have demonstrated that PCa cells-expressed PD-L1 are characterised by M2 macrophages, and this negatively correlates with deep changes of tumour inflammatory infiltrate composition including overexpression of PTX3, which appears to be an unfavourable prognostic marker [85]. Blockage of PD1 or PD1 ligands with pembrolizumab, nivolumab, lambrolizumab (PD1), atezolizumab and BMS-936559 (PD-L1) has demonstrated significant clinical anticancer activity by boosting T cell activation in multiple cancers including urothelial carcinoma. Pembrolizumab targets PD1 receptor by preventing it from binding to its immune-suppressing ligands, PD L1 and PD L2, therefore restoring robust T cell response that eradicate tumour cells [86]. A subset of HRPC that demonstrates dMMR pathway deficiency, a phenotype that is characterised by altered immune landscape, microsatellite instability, high mutation burden, an activated immune microenvironment, and increased PD1/PD-L1 expression on tumour and immune/stromal cells may also benefit from pembrolizumab [86,87]. McNeel et al. [30] has explored the antitumour activities of pembrolizumab and DNA vaccine encoding prostate acid phosphatase (PAP) when concurrently and sequentially combined in treating HRPC. This therapy elicited interferon-gamma (IFN-γ) secreting PAP-specific Th1-biased T cell immunity and CD8+ T cell infiltration with declined PSA only in concurrent therapy. Sadly this PSA positive response was reversed when the pembrolizumab treatment was stopped after 3 months. This suggested that the response was specifically related to the development of immune response from combination therapy of vaccination and pembrolizumab that usually targets dMMR, which were not the case in analysed patients [88].

The loss of biallaelic CDK12 is another important phenomenon and hallmark of MMR pathway deficiency in selected HRPC cases. This is usually characterised by increased gene fusions, which serve as neoantigens and promote intratumoral T cell infiltration that can potentially be targeted with pembrolizumab [63,89]. It is only 7% of HRPC patients that exhibit this genomic aberration, which means a one-size-fits-all approach that is currently being used must be reviewed and changed to specialised treatment. Developing inhibitors targeting these genomic aberrations may allow us to emulate current lung cancer model, in which the 5% to 6% of patients with non–small cell lung cancer who have an ALK rearrangement are treated with an ALK inhibitor [90,91]. This can be combined with checkpoint inhibitors and other approved HRPC treatments like sipuleucel-T to induce a favourable clinical outcome. A recent study has shown that the use of bipolar AT and enzalutamide has enhanced the response of mHRPC to anti-PD1 blockade, and this was associated with inactivation of mutations harboured by homologous recombination DNA repair genes. This suggested the therapeutic potential of IC blockade in patients with advanced PCa especially following immune activation [92]. Sena et al. [93] recently demonstrated a high clinical response rate in patients with deficient MMR after treatment with anti-PD1 pembrolizumab. Pembrolizumab resulted in prolonged progression free survival, OS and density of CD8+ tumour-infiltrating lymphocytes. These were strongly associated with tumour frameshift mutations, suggesting a new biomarker of ICIs sensitivity [93]. Nonetheless, the durability of the treatment response in some patients has been reported [93].

Unfortunately, ICI monotherapy has shown very minimal anti-cancer activity in PCa patients due to various disease factors including immunologically “cold” tumour microenvironment with poorly differentiated and few tumour infiltrating T cells. Dual combination of different ICIs or combining ICI monotherapy with currently approved HRPC hormone therapies such as enzalutamide significantly improves the efficacy of ICIs. Enzalutamide is an anti-androgen that reduces prostate tumour growth in HRPC cases by preventing the AR signalling pathway and transcriptional activities that feed tumour cells with testosterone [94]. In two separate studies including the CheckMate 650, treatment of abiraterone plus prednisone pre-treated and chemotherapy naïve HRPC patients with dual combination of enzalutamide and pembrolizumab showed a better objective response rate (ORR) with reduced PSA [78,95,96]. Graff et al. [27] recently showed that adding pembrolizumab treatment in HRPC patients progressing on enzalutamide alone induced a better clinical outcome with an objective radiographic response and PSA decrease of ≥50% as compared to HRPC patients on progressing enzalutamide monotherapy. Patients whose tumours exhibited no dMMR, CDK12 loss or PD-L1 expression also benefited from treatment in this trial [27].

Dual combination of nivolumab and ipilimumab has also demonstrated an ORR of 25% in chemotherapy-naïve HRPC cohort as compared to a HRPC cohort that has undergone chemotherapy (ORR of 10%). Here, ipilimumab turned a “cold” PCa tumour to “hot” by bringing in T cells to the tumour but simultaneously activated unneeded PD-L1, and this was blocked with nivolumab that intercepted PD1/PD-L1interaction and therefore freed the T cells to attack tumour cells. Although this combination provides a better clinical outcome, poor tolerability has still been reported and warranted for further research investigation [27].

### 3.3. V-Domain Immunoglobulin Suppressor of T Cell Activation

VISTA is a well-established immune regulatory receptor that can also serve as a ligand [97]. It is primarily and highly expressed in tumour infiltrating lymphocytes including in microglia, leukocytes, naïve CD4+ and Foxp3+ Tregs [98,99]. Although mechanisms underlying immunosuppressive role of VISTA are yet to be determined, VISTA has been naturally upregulated in tumour microenvironment of various malignancies such as leukaemia and pancreatic cancer. It has also recently been identified as an immunotherapy target of PCa owing it to its increased expression level in response to ipilimumab [100]. Ipilimumab-mediated expression of VISTA is directly proportional to increased expression of immuno-suppressive PD1 and PD-L1 in PCa, suggesting a compensatory inhibitory IC pathway that inspires novel effective combination-treatment strategies. A combination of ipilimumab and an anti-VISTA drug may offer a better clinical outcome in PCa [100].

### 3.4. Indoleamine 2,3-Dioxygenase (IDO)

Mesenchymal stem cells (MSCs) are known to play a pivotal role in tissue regeneration, wound healing and immune system, and have also been implicated in PCa development. MSCs have been demonstrated to promote transformation of androgen-dependent PCa into an androgen-independent tumour [101]. The co-culturing model of MSCs with tumour infiltrating lymphocytes has demonstrated that MSCs polarised to a Th1-like phenotype that was associated with marked pro-inflammatory changes [102]. Interferon-gamma (IFN-γ) and tumour necrosis factor-alpha (TNF-α) are two important factors that were produced by activated T cells in MSC polarisation, and this was associated with an upregulation IDO1 [103]. IDO1 catalyses the first and rate-limiting step of kynurenine pathway that converts L- tryptophan into the immunosuppressive metabolite L-kynurenine. It was also found that IDO1 is activated in some antigen-presenting cells in various tumours by tumour, MSCs and innate immune cells. IDO1 suppresses CD8+ T effector cells and natural killer cells as well as increased activity of CD4+ Tregs and MDSC. This influences immune tolerance to tumour antigens and evasion from immune-mediated destruction, which are significantly associated with poor prognosis. Previously, an increased expression of IDO1 correlated with high levels of PCa candidate biomarkers AMACR A, TNF-β1 and kynurenine in a subset of PCa patients [104]. Patients with advanced PCa exhibit an increased activation and expression of IDO1 after treatment with either DNA vaccine PAP or/and anti-PD1 inhibitor pembrolizumab. There was no PSA response as confirmed by lack of PSA decline following treatment. However, an induction of specific IFNγ-secreting T cell response was observed following in vitro stimulation of peripheral blood cells with 1-methyltryptophan that inhibit IDO. Given this data, activation and expression of IDO1 appears to be an underlying mechanism of immune evasion used by PCa. Counteracting IDO1 activities has resulted in the reactivation of anticancer immune responses in animal studies with tumour models [105], suggesting that blockage of IDO1 may also represent a promising therapeutic candidate for HRPC.

### 3.5. T cell Immunoglobulin Domain and Mucin Domain 3 (TIM-3)

TIM-3 is a member of Ig superfamily and is highly expressed on fully differentiated Th1 lymphocytes, CD11b+ macrophages, activated T and myeloid cells. TIM-3 regulates macrophage, activates and inhibits Th1 mediated immune responses to promote immunological tolerance. Increased level of TIM-3 expression was observed on both CD4+ and CD8+ T cells in PCa patients and this correlates with a higher Gleason score (>7) and increased pre-operative PSA [106]. On the contrary, reduced TIM-3 expression was associated with poor prognosis in metastatic PCa and served as a biomarker to differentiate metastatic HRPC from mHSPC, making the prognostic value of TIM-3 in PCa controversial [107].

Zhang et al. [108] has recently conducted preclinical studies in mice models with PCa tumours and demonstrated that triple therapy of streptavidin-GM-CSF surface-anchored tumour cell (anchored GM-CSF) vaccine, anti-TIM-3 and anti-PD1 antibodies in sequential pattern inhibited tumour growth and increased tumour regression rate in more than 60% of tested mice. This was significantly a better clinical outcome compared to concurrent therapy of anchored GM-CSF vaccine and PD1 inhibitors that, although induced robust antitumour activities, was ultimately associated with aggressive tumour progression and minimal regression in some mice. This supports numerous previous studies that demonstrated that TIM-3 is co-expressed on dysfunctional or exhausted T cells with PD1 as a compensatory and synergistic partner that, when co-blocked with PD1, reverses immune resistance in preclinical studies and restores anticancer T cell responses in patients with advanced cancer [109,110,111].

Galectin-9 (GAL9) is a TIM-3 ligand that belongs to the galectin family of lectins. Interaction of TIM-3 with GAL9 induces T cell dysfunction and predicts poor prognosis in patients with other solid tumours [112]. In PC-3 PCa cells, GAL9 was found to induce atypical ubiquitination leading to non-apoptotic cell death [113]. High-mobility group box 1 (HMGB1) and phosphatidylserine (PS) are two other ligands for TIM-3 with immunosuppressive roles in infections and various malignancies [114,115]. There may also be possible activation of TIM-3/GAL9 signaling pathway in PCa, and this still warrants research investigation to determine the effect of this pathway in PCa cell transformation and clinical outcome.

### 3.6. Lymphocyte-Activation Gene 3 (LAG-3)

LAG-3 is expressed on B cells, natural killer cells and plasmacytoid dendritic cells, but also constitutively and predominantly on activated cytotoxic T cells and Tregs. LAG-3 normalises both innate and adaptive immune responses by limiting cytokines secretion, T cell activation and proliferation leading to their exhaustion. Its main ligands are major histocompatibility complex class II (MHC-II) molecules, which are constitutively expressed on professional immune APCs. LAG-3 is structurally similar to CD4 receptor and both bind to antigen MHC-II as their canonical ligand (Figure 2a). However, LAG-3 binds MHC-II with 100-fold higher affinity than CD4 and negatively regulates proliferation, activation, and homeostasis of T cells in a similar fashion to CTLA4 and PD1 [114]. In the tumour microenvironment, LAG-3 becomes upregulated on Tregs, gathers around tumour sites and exerts immunosuppressive roles that amplify dysfunctional cytotoxic T cells and induction of deficient antitumour immune response. Enrichment of LAG-3 and CD8+ T-cells expressing tumour-infiltrating lymphocytes with better clinical outcome was observed in estrogen receptor negative breast cancer, indicating an independent prognostic value [116]. Indeed like TIM-3, LAG-3 can be co-expressed with other ICIs like PD1 as observed in preclinical mouse tumour models and cancer patients with intratumoral T-cell dysfunction that accentuates immune escape and increased tumour growth. This led to perturbed TNF and IFN-γ inflammatory signaling pathways which were restored with dual combination therapy counteracting both LAG-3 and PD1 activities [117,118,119,120,121]. Dual inhibition of LAG-3 and other ICIs synergistically increases T cell tumour anti-activities.

Upregulation of LAG-3 by tumour-specific CD4+ and CD8+ T cells in Tregs infiltrating PCa lesions has been observed. A contrary finding of a rather low LAG-3 expression was reported in various studies [53,122,123], suggesting that further research investigations are needed on this subject. LAG-3 also interacts with galectin-3 (GAL3) and liver sinusoidal endothelial cell lectin expressed on tumour cells and tumour-associated stromal cells, respectively. GAL3 was demonstrated to exert two opposite physiological roles based on its cellular localisation in PCa. Nuclear GAL3 may promote antitumour activities while cytoplasmic GAL3 may enhance tumour aggressiveness [124], and this may explain the contradictory level of LAG-3 expression status in the two studies. Significant reduction of nuclear GAL3 following its promoter hypermethylation has been observed in PCa, and this correlates with disease progression [125]. It has also been shown that activation of GAL3 in PCa cancer cells and xenograft mouse models with PCa causes the induction of T cell apoptosis, angiogenesis and bone metastases, which were pharmacologically reversed with RNA interference or interventions counteracting the activities of GAL3 [126,127,128,129]. Other studies have also shown that cleavage of GAL3 by metalloproteinase 2, 9 and PSA favors tumour progression in PCa suggesting its potential for therapeutic targeting [129,130]. Moreover, GAL3 activation upregulates the AR and its downstream target genes, hence, its implication in the resistance to enzalutamide and bicalutamide anti-AR drugs in xenograft mouse model [126,128].

### 3.7. T Cell Immunoreceptor with Ig and ITIM Domains (TIGIT)

TIGIT is also TIGIT is also known as CD223, WUCAM and VSRM3. It is a T cell and NK-cell expressed immune receptor that interacts with CD122 (Nectin-2), other nectins and CD155 (PVR) on DCs and macrophages to convert inhibitory signals on immune system [131,132,133]. TIGIT has a greater affinity to its ligands, enabling it to overcome its competitor CD226 to bind CD122 and CD155. Dysregulation of TIGIT allows tumour cells to upregulate CD122 and CD155 and avoid immune-mediated destruction. TIGIT functions like PD-L1 and, when blocked, it enhances T cell proliferation and function [134]. Co-inhibition of TIGIT with anti-TIGIT antibody tiragolumab and anti-PD-L1 antibody atezolizumab restored functional tumour-specific T cells leading to greater ORR and progression-free survival in patients with non-small lung cancer. Papanicolau-Sengos et al. [135] has demonstrated over-expression of CD122, CD155 and CD276 (B7-H3) that differentiated HRPC from HSPC, representing another potential target for aggressive PCa immunotherapy.

### 3.8. B7 Homolog 3 (B7-H3)

B7-H3 B7-H3 is also known as CD276, a member of the B7 family of the IgSF [136,137]. It is predominantly expressed on professional APCs including B cells, macrophages, DCs and a wide variety of tumour cells [138]. It is also expressed on a lower level in a broad variety of non-immune cells, suggesting additional non-immunological functions [136,137,138]. Both stimulatory and inhibitory properties have been identified but the ligand of B7-H3 has yet to be identified. Like TIGIT, B7 homolog 3 (B7-H3), a member of the B7 family of the IgSF, is similar to PD-L1. B7-H3 is upregulated in PCa where it is negatively correlated with biochemical cancer recurrence, progression and metastasis [139]. Several studies have revealed increased B7-H3 expression that correlates with clinicopathologic indicators of aggressive cancer, metastasis and poor clinical outcomes in PCa patients [140,141,142,143]. Moreover, expression of B7-H3 tends to be related to androgen signalling and immune reactivation [135,141,143].

Enoblituzumab is a humanised Fc-optimised B7-H3–targeting antibody that induces antibody-dependent cellular cytotoxicity (ADCC). Treatment with this monoagent conferred antitumour activities in both localised intermediate and high-risk PCa in Phase II clinical studies [144]. Phase I study of anti-B7-H3 bispecific antibody in PCa patients enhanced T cells activation and proliferation as well as production of cytokines and mediators (granzyme/perforin) that eliminates tumour cells, qualifying B7-H3 a potential target for PCa immunotherapy [145].

### 3.9. V-Set Domain-Containing T Cell Activation Inhibitor 1 (VTCN1)

VTCN1, also known as B7 Homolog 4 (B7-H4), is a glycosylated member of the B7 family that delivers costimulatory signals [146]. However, VTCN1 negatively regulates T cell-mediated immunity and potentiates immune evasion, epithelial cell transformation, proliferation, cytokine production and the development of cytotoxicity by suppressing T cell activation in the tumour microenvironment. Prostate, liver, kidney, lung, spleen, pancreas, placenta, testis and thymus are tissues that primarily express VTCN1 [68]. Upregulation of VTCN1 has been observed in various tumour tissues, and this is parallel with poor clinical and tumour aggressiveness pathological features [147,148]. In PCa, an elevated level of VTCN1 is associated with activation of genes that establish cancer stem cells (CSC), pathological high tumour stage and poor or shorter OS rate, making it a potential independent prognostic biomarker and therapeutic target [149,150]. CSC, a hallmark of advanced PCa, are a heterogeneous population of tumour cells with the capability for self-renewal, and are associated with increased motility that enables tumour invasion and metastases leading to PCa therapy resistance [151,152,153].

VTCN1 increased expression level is associated with a higher incidence of PD-L1 co-staining. Thus, therapeutic co-blockade of B7-H4 and PD-L1 could favourably alter the tumour microenvironment allowing for antigen-specific clearance of tumour cells [154]. Blockage of the interaction between VTCN1 and its receptors overcomes tumoral immune escape and correlates with increased T cells and NK cell infiltration that suppresses PCa tumour growth and metastasis [155,156].

## 4. Nuclear Medicine: Peptide Receptor Ligand Therapy

Chemotherapy and radiation have a disadvantageous trade-off of reduced physical activity (PA) and quality of life (QoL), and are associated with severe side-effects [157]. PRLT with radionuclides are rapidly emerging as a better alternative and cost-effective treatment option for managing advanced PCa. It promotes patient-centred care and personalised treatment, meaning the right drug is administered to the right patient at the right time. This will speed up the time taken to find an effective treatment for patients and saving them the costs of drugs that are likely to be ineffective. Most importantly, PRLT therapy is non-invasive and runs only for few months with increased efficacy and minimal cycles and toxicity [158,159]. PRLT is delivered systemically without damaging any healthy tissues or cells hence it has been labeled as a safe and effective treatment.

**Figure 4:**
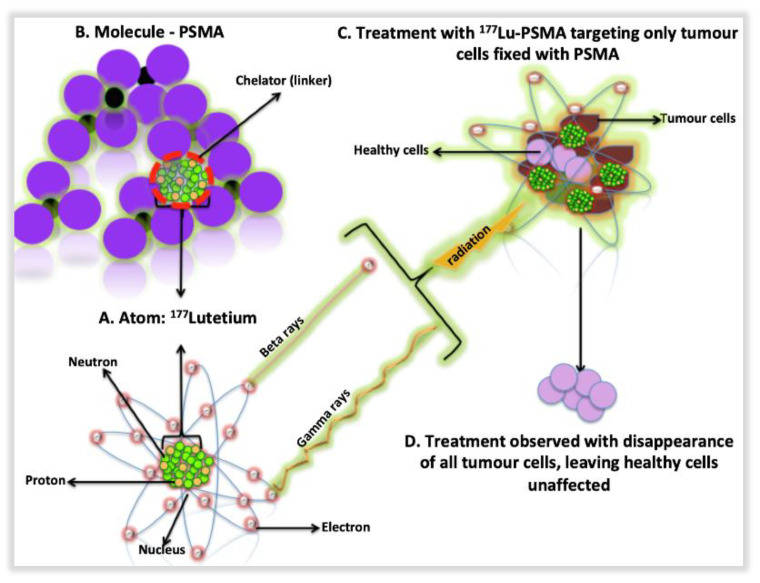
Radionuclides-based therapy pathway and killing of tumour cells through targeted alpha therapy using ^177^Lutetium-PSMA as an example. (**A**) The radionuclide (e.g., ^177^Lu-PSMA) is the radioactive isotope that releases energetic particle radiation such as α or *β* particles for therapeutic purpose. (**B**) The second part is a small peptide that is designed to bind to the cellular target of interest. The third part is a linker molecule that serves to connect the radionuclide with the leading peptide. In PRLT of PCa, ^177^Lutetium, a *β* particle-emitting radionuclide, is the most commonly radionuclide that is complexed to a PSMA ligand using a linker molecule. Following the intravenous administration of ^177^Lu-PSMA, the complex easily permeates into tumor foci with the smallness of the complex facilitating efficient penetration into the tumour core. (**C**) Following binding to the extra-cellular component of the PSMA receptor expressed on the tumor cell membrane, ^177^Lu-PSMA-receptor complex undergoes internalization into the tumor cell cytoplasm. (**D**) This allows radiation delivery to occur near the tumor nuclei making tumor cell killing more efficient. In addition to *β* particle emission, ^177^Lutetium also emits γ photons (for diagnostic purpose) that can be used for imaging using a single photon emission tomography (SPECT) imaging technique. The ability to perform imaging following the administration of ^177^Lu-PSMA for PRLT allows for the determination of the in vivo distribution of the therapy agent and the confirmation of the uptake of the treatment agent at the tumor sites. In addition, imaging the γ photons of ^177^Lutetium allows for the determination of the radiation dose delivered to the tumor foci (dosimetry).

PRLT is an integral part of nuclear medicine and is applied in the setting of a theranostics platform, which incorporates both therapy and diagnostics. It involves the use of radionuclides (also known as radioisotopes or radiopharmaceuticals) that typically undergo radioactive decay and lose energy in the stochastic process to produce another stable or unstable daughter nuclide. These radionuclides are usually conjugated to vectors such as peptides, nanoscale and monoclonal antibodies that are capable of delivering cytotoxic radiation therapy directly to and treat the targeted primary malignancies, bone and visceral metastases (Figure 4). They usually emit either beta (β) (e.g., ^177^Lutetium: ^177^Lu) or alpha (α) (e.g., ^213^Bismuth: ^213^Bi, ^225^Actinium: ^225^Ac and ^223^Radium: ^223^Ra) atoms. Gamma (γ) and positron-emitting radionuclides are used for diagnostic purposes using Single Photon Emission Computed Tomography (SPECT) or PET imaging techniques [160,161].

The new emerging PRLT in advanced PCa are directed towards PSMA, a type-II transmembrane glycoprotein belonging to the M28 peptidase family. PSMA is widely used to guide the current radioisotopes during PCa treatment, owing to its over expression regardless of treatment in PCa especially metastasis [162,163]. Some tissues including salivary glands, small intestine and renal tubules may also express PSMA but at a very minimal level, making PSMA a good target for several nuclear medicine imaging and therapeutics in advanced PCa [164]. The advantage of using PRLT as a theranostics approach to managing mHRPC is that pre-therapy, i.e., initial staging, restaging, monitoring and follow-up assessment and detection of recurrent disease with ^68^Ga-PSMA PET/CT imaging can be performed, and the lesions can also be objectively assessed by providing a reproducible data. The proPSMA study by Murphy and Hofman has elegantly demonstrated that novel imaging with ^68^Ga-PSMA-PET/CT was significantly accurate to determine the extent of lymph nodes or distant disease that was not apparent on standard or conventional imaging using CT and bone scintigraphy or magnetic resonance image [165]. In addition, ^68^Ga-PSMA PET/CT was demonstrated to be far superior to conventional bone scintigraphy in the detection of PCa related skeletal metastatic lesions; this finding has introduced a turn in the manner in which clinically bone metastases are evaluated [166]. Additionally, critical in the management of PCa is the detection of BR after radical prostatectomy, this was demonstrated in a retrospective study [167]. Furthermore, Calais et al. [168] broadened the indications of ^68^Ga-PSMA PET/CT imaging to include a role in SRT planning; this was the first prospective trial of its kind to demonstrate the clinical utility of this modality to improve patient outcomes. In intermediate risk PCa males who may benefit from additional pelvic lymphadenopathy dissection, further imaging with ^68^Ga-PSMA-11 PET/MR has been shown to yield additional detailed anatomical information as reported by Park et al. [169].

^177^Lu-PSMA is a β-particle emitting radionuclide that has been extensively investigated and still receives prominence in treating and managing the PCa [170]. Baum et al. [171] have observed more than 80% PSA decline rate and reversible short-term side-effects in patients underwent ^177^Lutetium-labeled DOTAGA-based PSMA ligand and ^177^Lu-DOTAGA-(I-y)fk(Sub-KuE) (^177^Lu-PSMA) radiotherapies. Hofman et al. [165] have also shown that ^177^Lu-PSMA is safe, efficacious and well-tolerated with minimal side-effects including reversible xerostomia and a minor reduction in erythrocyte count. A recently published multicenter analysis has demonstrated a significantly longer OS following [^177^Lu]Lu-PSMA-617 therapy (14.6 months) in chemotherapy-naïve mHRPC patients than patients with a history of chemotherapy with docetaxel or docetaxel followed by cabazitaxel (11.1 months) [172]. Several separate systemic reviews have further shown that ^177^Lu-PSMA-617 therapy provides better clinical benefit (37% OR, 38% stable radiographic disease and best PSA decline of ≥50%) with minimal side-effects in a subset of patients [173,174]. However, the best PSA decline of ≥50% and objective remission were observed with only ^177^Lu-PSMA-RLT than when it was followed by the third-line treatment with enzalutamide and cabazitaxel which led to discontinuation of treatment because of adverse reactions [173]. The efficacy of radioligand therapy with ^177^Lu-PSMA-617 in managing mHRPC patients is influenced by various factors such as age, liver enzymes, haemoglobin, Gleason score, platelets, C-reactive protein (CRP) and pain medication dependency [175,176]. A higher lactate dehydrogenase (LDH) has a negative impact on the therapeutic response; however, the multivariate analysis revealed that the most significant independent factors were the number of platelets and regular need for pain medication. Rational combination clinical trials of ^177^Lu-PSMA-617 with other treatment modalities including the use of PARP inhibitors, immune-oncology agents and antibody-based radioligand therapy targeting PSMA are also possible, i.e., ongoing PRINCE and LuPARP trials.

Several PSMA ligands, e.g., J591 (a monoclonal antibody that targets the extracellular domain), PSMA-617 and PSMA-I&T have also been used with varying success in the endotherapy of PCa [177]. Kratochwil et al. [178] also highlighted the important usage of theranostics in which ^177^Lu-PSMA RLT was utilised to induce remission in a patient with metastatic PCa.

Despite the promising results with PRLT using ^177^Lu-PSMA-617, some patients still demonstrate disease progression assessed by PSA progression or appearance of new lesions on imaging with either ^68^Ga-PSMA PET or ^99m^Tc-PSMA SPECT. This opened a new therapeutics avenue such as the usage of targeted alpha-emitting radionuclides like ^225^Ac, ^213^Bi and ^223^Ra. Recently, our group reported an initial pilot project on the use of ^225^Ac-PSMA-617 in South African chemotherapy-naïve patients with advanced PCa. Although the sample size was modest, the breakthrough was the demonstration of decrease in disease-burden that was associated with the decline in sPSA [179]. The most debilitating side effect was xerostomia. It was purported that a de-escalation of the activity in subsequent cycles might improve the safety profile of this therapy. Kratochwil et al. [178] have observed ≥50% PSA decline and PSA decline of any degree in 63% and 87%, respectively, of mHRPC patients treated with ^225^Actinium-PSMA-617. Xerostomia was the main adverse reaction leading to treatment discontinuation in 4 patients, suggesting further modification of the treatment regimen to increase efficacy. Sathekge et al. [40] demonstrated that restaging of ^213^Bi-PSMA-617 after 11 months benefited mHRPC patients by yielding a remarkable imaging response and reduced PSA level (from 237 μg/L to 43 μg/L).

Nonetheless the above data, there are few setbacks that led to discontinuation or further studies with ^213^Bi. ^213^Bi is a mixed α/β emitter with an extremely short half-life of 46 min and mainly decays via beta- emission to the ultra-short- lived pure alpha emitter ^213^Po. Morgenstern et al. [179] demonstrated the usefulness of targeted alpha therapies; however, worldwide supply and production still remains a significant challenge in order to promulgate these strategies as routine in the clinical setting. In our clinical setting, ^213^Bi is rarely used due to the sporadic production, clinical availability and physical characteristics that potentially limit efficacy in skeletal metastases. The clinical use of α-emitters is gaining popularity together with the list of available armamentarium. Müller et al. [180] has described the power of targeted therapy with other α-particle emitters, namely, ^211^At, ^225^Ac and ^213^Bi, and this provided promising data especially when used in combination with tumour-targeted antibodies.

^223^Ra-dichloride/Xofigo^®^ is another alpha-emitting radionuclides with potential effects in treating advanced PCa patients. It is now widely in use for PCa with skeletal metastases. It has received approval from the FDA (2013), EMA (2013) and SAHPRA (2016). Parker et al. [181] conducted a Phase III trial, i.e., the ALSYMPCA (ALpharadin in SYMptomatic Prostate Cancer) Trial which demonstrated the safety, efficacy and improved OS [Hazard ratio 0.7] in patients with mHRPC with or without prior use of chemotherapy with docetaxel while on an alpha-emitter ^223^Ra-dichloride. Another study has shown that optimum dosage of ^223^Ra-dichloride and alkaline phosphatase exhibited longer OS rate and time to first skeletal-related events (SREs) in mHRPC patients. Additionally, limited rates of both hematological and non-hematological adverse reactions were observed in bone metastases [182,183,184]. NCT03093428 and NCT02463799 trial studies with the use of ^223^Ra with or without atezolizumab and sipuleucel-T in mHRPC patients are currently ongoing and the data is blatantly awaited (Table 1).

## 5. Evidence-Based Support for Combination of ICIs and PRLT

The combination of PRLT and ICIs immunotherapy may improve tumour regression and OS in patients with advanced PCa disease. The current FDA approved agents available for treating PCa (docetaxel, sipuleucel-T, cabazitaxel, abiraterone, enzalutamide, Xofigo, zoledronic acid, denosumab and apalutamide/darolutamide) are highly expensive with a cost ranging from 3000–100,000 USD/cycle or month [185], and yet they still fail to yield a universal prolonged progression-free survival or higher objective response rate. An increase in OS conferred on patients with mHRPC treated with the currently approved agents last for only a few months [32,77]. A need, therefore, exists for the continued improvement in treatment options for advanced PCa. One strategy for improving the outcome of advanced disease treatment is by the rational combination of therapeutic agents acting on different pathways within the tumour. Such combinatorial therapy must have no overlapping toxicity and able to establish safety and universal treatment efficacy.

In certain clinical settings, the standard of care (SOC) is chemotherapy, notably docetaxel in combination with ADT in both mHSPC and mHRPC. There are no incremental benefits noted in docetaxel when added to other treatment strategies like DNA vaccines and ICIs [12]. This may be the same case with ADT in combination with older agents such as mitoxantrone due to the differences in epigenetics and genetic mutations (e.g., dMMR) profiles between different population groups [186]. PRLT has proven to be an effective treatment in advanced diseases where standard treatment (ADT, immunotherapy or chemotherapy) is no longer effective and offset by complications and side effects. Most importantly, PRLT can target the disease in the mHRPC setting, the fatal form of PCa. Despite the success rates in the treatment of other types of cancer, ICIs have offered insignificant clinical benefit in the treatment of advanced PCa. However, concurrent rather than sequential combination of radionuclide therapies (e.g., ^223^Ra-dichloride and^177^Lu-PSMA-617) and ICIs immunotherapies from early on may synergistically improve anti-cancer efficacy of ICIs and ultimately QoL, long-lasting durability and OS rates while mitigating various complications associated with monotherapy or standard treatment. A recognized rationale for the limited response rate of mHRPC to ICIs is the relatively low neoantigen loads seen in prostate tumors [187]. A high ICIs-mediated tumour antigen release here exposes tumour cells that initially evaded from immune surveillance to be destroyed by robust restored cytotoxic T cells. Sena et al. [93] recently demonstrated that tumour frameshift mutation proportion offered more robust neo-antigenic peptides that were targeted by anti-PD1 antibody pembrolizumab leading to a better clinical response in dMMR PCa patients [93]. Unfortunately, durability of the treatment response in a subset of patients remains a big challenge, suggesting the need to boost the strength and longevity of the treatment.

Mutations in DNA-damage repair and checkpoint genes were detected in mHRPC patients who are resistant to PSMA-targeting alpha radiation therapy (TAT) despite PSMA-positivity, suggesting a combination therapy of PSMA-TAT with PARP inhibitors and ICIs [178]. This hypothesis was supported by a recent published study by Ravi and Hofman [188]. Czernin et al. [189] have also supported this proposal by showing that ICIs targeting PD1 enhanced the efficacy of ^225^Ac-PSMA-617 in PCa mouse model. This observation may be explained by endotherapy with PRLT. This usually causes sustained tumour destructions over days leading to a protracted release of tumour antigen into the circulation. The released tumour antigens activate host immune system, which may lead to the enhancement of the abscopal effect—the effect described following radiotherapy in which tumour shrinkage occurs at non-irradiated tumour sites remote from foci of irradiated tumour. The abscopal effect is hypothesized to result from the generation of systemic antitumour response following radiation-induced immunogenic tumour cell death [190]. The combination of PRLT with ICIs may increase the neoantigen release from the PCa tumour augmenting the immune activation caused by ICIs thereby leading to improved efficacy. This combination therapy is currently being tested in the ongoing PRINCE (PSMA-lutetium Radionuclide Therapy and ImmuNotherapy in Prostate CancEr) trial (NCT03658447) where the safety, tolerability, and efficacy of the combination of ^177^Lu-PSMA and pembrolizumab (a PD1 inhibitor) will be assessed in patients with mHRPC. In the single arm Phase Ib/II trial, 200 mg pembrolizumab given 3 weekly for up to 35 cycles will be combined with up to 6 cycles of 8.5 GBq of ^177^Lu-PSMA given 6-weekly.

Nanoparticles have been explored for specific delivery of therapy agents to tumour foci. This targeted delivery of therapy agent helps increase drug concentration in the tumour while reducing off-target effect thereby reducing the incidence and severity of therapy—Induced toxicities. The potential benefits and challenges associated with incorporating radionuclide agents such as alpha emitters like ^223^Ra and ^225^Ac have been recently reviewed by Czerwińska and colleagues [191]. This treatment intervention holds promise to reduce the severity of toxicities associated with radio-ligand therapy of PCa while increasing the dose delivered to the tumour.

## 6. Conclusions

Theranostics is currently a revolutionary diagnostic and treatment modality for PCa especially mHRPC, which is difficult to treat. Incorporating theranostics as a combination treatment modality in PCa patients may improve the efficacy and safe profiling of immune-based therapies. We believe that there is unequivocal clinical trial evidence that strongly supports and suggests that the most useful approach in the management of patients with PCa would be with combination therapies; in order to risk stratify the patients and mitigate the complications and side effects. PCa is notoriously heterogeneous, and one size fits all approach has not shown to be effective because every patient responds differently to the disease and treatment. The more complex the PCa disease is, the more personalised treatment we need. The operational framework would aim to improve survival rates among patients in different disease stages and comprise of multidisciplinary approach with the geneticist, urologist (surgery), nuclear physician, pathologist, radiologist, oncologist and palliative care. Radionuclide therapy is currently a revolutionary theranostics approach for advanced PCa, which is usually difficult to treat. Integrating radionuclides and ICs therapy into one platform from early on may shift the paradigm of PCa treatment by leading both to the direct destruction of the tumour and recruitment of immune system against the tumour. It is, however, imperative that therapeutic protocols or guidelines be designed to determine the best dosage combination of these therapies taking into consideration disease histology pattern, optimal timing, side-effects and sequencing. The main limitation of radionuclide therapy in treating PCa is having a universal marker of the disease. Most radionuclide therapies use PSMA as a peptide, and unfortunately, this is not universally expressed by all PCa tumour cell subpopulations. More studies are needed to verify the consistency of the expression of other potential markers such as IDO1, LAG-3/GAL3 and H7-B3 ICs in PCa to determine whether they could qualify as additional markers for metastasised PCa-targeted and personalising therapeutic approach. This way several pathways can be counter targeted eliminating the unnecessary treatment of patients in whom standard PCa treatment is inappropriate. Additionally, this may help to optimise drug selection for a particular patient leading to effective treatment and prolonged QoL and OS of patients in our clinical settings.

## Figures and Tables

**Table 1:** Summary of key trials and studies in the treatment of PCa (2006–2020).

Trial Names	Design	Results/Endpoints	Complication/S	Comment
ADT ALONE OR IN COMBINATION				
**NCT00309985** **(CHAARTED)** [16]	Interventional: Clinical Trial (Randomised) Prospective	• OS • Rate of PSA level less than 0.2 ng per millilitre	• Fatigue, neutropenia and allergic reactions were noted in patients combination therapy	• 6 cycles of docetaxel + ADT vs. ADT alone • 13.6 months increase in group on combination therapy (docetaxel + ADT) • Rate of complications was higher in combination group, i.e., 6.2% vs. 0.5%
**NCT00268476** **STAMPEDE** [17]	nct00007644 Prospective	• OS + FFS	• Cardiovascular and renal toxicities	• Highlighted importance of combination therapies and prospective trials
**NCT00055731** **GETUG-12** [18]	Interventional: Clinical Trial (Randomised) Prospective	• Relapse-Free Survival • OS	• Cardiovascular disease	• ADT alone vs. supported combination therapies of ADT + radiotherapy + estramustine
**NCT00667069** **GETUG-AFU 17** [19]	Interventional: Clinical Trial (Randomised; Multi-centre)	• OS • MFS + • Acute/chronic toxicity • QoL	• Genitourinary toxicity and erectile dysfunction (ED)	• Phase III; Efficacy of triptorelin (ADT) + RT soon after surgery compared to surgery in localised PCa • GETUG-AFU 17 show that a RT significantly increased the risk of late toxicity • Randomised Phase 3 trial, Ongoing, to be completed in 2022
**SASCRO/SASMO CONFERENCE ORAL PRESENTATION (BOSHOMANE ET AL., 2016-UNPUBLISHED DATA)**	Prospective	• Response & safety	• No side-effects & adverse presented	• Demonstrated favourable response, i.e., no lesions and also a decrease in sPSA levels.
**NCT00007644** **PIVOT** [20]	Interventional: Clinical Trial (Randomised) Prospective	• All-cause mortality & PCa related mortality	• Adverse events presented (one fatality)	• Radical prostatectomy/surgery did not reduce PCa related mortality rates, i.e., disease still progressed
**REGISTRY NUMBER 12615000912583** **NEW ZEALAND TRIAL** [21]	Prospective	• Response to therapy according to PCWG criteria and QoL	• Used 7.5 GBq mean dose per cycle, reported mainly xerostomia	• Phase II trial in mHRPC in patients who progressed on ADT and chemotherapy
**NCT01715285** **LATITUDE TRIAL** [22]	Interventional: Clinical Trial (Randomised)	• OS and radiographic disease progression	• Grade 3 adverse events were more in the abiraterone group	• Exclusively enrolled men with high-risk mHSPC and excluded previous chemotherapy •Addition of abiraterone acetate and prednisone to ADT significantly increased OS and rPFS in men with newly diagnosed, metastatic and castration-sensitive prostate cancer
**NCT02677896** **ARCHES TRIAL** [23]	Interventional: Clinical Trial	• OS • Enzalutamide (second-generation nonsteroidal antiandrogen) + ADT (AR inhibition) significantly reduced risk of metastatic progression or death overtime vs. placebo plus ADT in men with mHSPC.	• Endocrine disorders • Gastrointestinal	• Enzalutamide + ADT may be considered in men with mHSPC (with low-volume disease) or who received prior docetaxel
**NCT02485691** **CARD CLINICAL TRIAL** [24]	Interventional (Clinical Trial)	• Measurement of rPFS (2 years-time frame) • Radiographical PFS as defined from randomisation time from the occurrence of death due to any cause	• Adverse event (Grade 3) were more in the cabazitaxel arm than the androgen-signalling-targeted inhibitor	• Preference may be given on cabazitaxel rather than the addition of another novel androgen receptor pathway inhibitor • Patients with mHRPC may benefit from PARP inhibition • Phase IV (4)
**RADIOTHERAPY**				
**RTOG 9902** **(RADIOTHERAPY)** [25]	Interventional: Clinical trial (Randomised, Phase III)	• Hypothesis—addition of combination chemotherapy to RT would increase OS	• Cardiovascular events • Trial stopped early because of thromboembolic toxicities	• Chemotherapy toxicity evident • No significant differences in OS, biochemical failure, local progression, distant metastases or disease-free survival with addition of adjuvant CT to LT AS + RT. • Long follow-up period ~10 years
**PARP-INHIBITORS**				
**NCT01085422** [26]	Interventional	• Pilot study combining an oral PARP with temozolomide in men with mHRPC	• Adverse events: thrombocytopenia, anaemia, fatigue, neutropenia	• Phase I (ABT-888, i.e., veliparib PHASE 1)
**IMMUNE CHECKPOINTS**				
**NCT03834493** **(KEYNOTE-641)**	Interventional: Clinical Trial (Randomized)	• OS • rPFS	• N/A	• Phase III, Pembrolizumab with Enzalutamide vs. Placebo with Enzalutamide in mHRPC patients • Trial is currently active and will be completed in 2023
**NCT03040791**	Interventional: Clinical Trial	• PSA response rate	• N/A	• Phase II, Nivolumab in PCa with DNA Repair Defects (ImmunoProst) • Trial is currently active and will be completed in 2021
**NCT03248570**	Interventional: Clinical Trial (Non-Randomized)	• ORR	• N/A	• Phase II, Pembrolizumab in mHRPC with or without DNA Damage Repair Defects • Trial is currently active and will be completed in 2023
**NCT03061539**	Interventional: Clinical Trial	• Radiological response • PSA response ≥50% • Conversion of CTCs from ≥5 to <5 cells/7.5 mL	• N/A	• Phase II, Nivolumab with ipilimumab in PCa with an immunogenic signature • Trial is currently active and will be completed in 2025
**NCT03570619** **(IMPACT)**	Interventional: Clinical Trial (Non-Randomized)	• ORR	• N/A	• Phase II, Nivolumab with ipilimumab in mHRPC with CDK12 Mutations • Trial is currently active and will be completed in 2021
**NCT03834493**	Interventional: Clinical Trial (Randomized)	• OSr • PFS	• N/A	• Phase III, Pembrolizumab with enzalutamide vs. placebo with enzalutamide in mHRPC patients (KEYNOTE-641) • Trial is currently active and will be completed in 2024
**NCT02601014** **(STARVE-PC)**	Interventional: Clinical Trial (Randomized)	• PSA decline >50% • Safety & Tolerability	• N/A	• Phase II, Nivolumab & Ipilimumab targeting AR-V7 in mHRPC • Trial is currently active and will be completed in 2022
**NCT02312557** [27]	Interventional: Clinical Trial	• Efficacy of pembrolizumab in men with mHRPC • Active, not recruiting	• Grade 2–5 toxicity: myositis, GIT and endocrine complications.	• Phase II • Pembrolizumab and Enzalutamide • Responders (18%) showed a decline of sPSA > 50%;
**NCT00323882** [28]	Interventional (Prospective)	• Completed	• GIT, liver, skin, eyes and endocrine glands.	• Phase I/II, • MDX-010 ± RT; Ipilimumab monotherapy & with RT • FINAL DATA PENDING
**NCT01498978** [29]	Interventional: Clinical Trial	• Completed	• No evidence of toxicity	• Phase II, Ipilimumab monotherapy and with ADT
**NCT00702923** [30]	Interventional Unregistered (sponsored)	• Terminated due to slow accrual	• Gastrointestinal	• Phase I • Tremelimumab monotherapy and with ADT (bicalutamide) • Three of 11 experienced delayed PSA doubling time
**NCT00113984** [31]	Interventional: Clinical Trial	• Safety and tolerability of combination of fixed dose of vaccine & vaccine & anti-CTLA4	• No immune-related adverse effects noted	• Phase I; PSA-targeted vaccine that enhances co-stimulation of the immune system did not seem to exacerbate the immune-related adverse events associated with ipilimumab
**NCT00861614** [32]	Interventional: Clinical Trial	• PFS & OS • Primary end points not met.	• GIT (Diarrhea, vomiting, nausea, reduced haemoglobin, headaches and dizziness.	• Phase III • Ipilimumab following RT • OS (10 months vs. 11.2 months), PFS (4 vs. 3.1 months with HR 0.70; *p* < 0.0001
**PEPTIDE RECEPTOR LIGAND THERAPY**				
**NCT03392428** **(ANZUP PROTOCOL 1603)** [33]	Interventional: Clinical Trial (Randomized)	• Primary endpoints: PSA response rate, PFS, QoL	• Data not published yet	• Phase II; • ^177^Lutetium-PSMA-617 vs. Cabazitaxel in mHRPC • Ongoing—Estimated completion date January 2021 and the results are eagerly awaited
**NCT01106352** **ALSYMPCA TRIAL** [34]	Interventional: Clinical Trial (Randomized) Prospective	• OS improved	• Febrile neutropenia	• Phase III trial using alpha radiation particles • 50k Bq/kg intravenous 4 cycles • Comparison with SOC (chemotherapy, e.g., docetaxel)
**REVIEW ARTICLE** [35]	Review article	• Safety & Efficacy, OS, PFS, FFS and QoL	• Grade ¾ hematologic toxicities; thrombocytopenia; anemia, pyrexia, back pain, fatique, etc.	• All available therapeutic agents • Change of landscape in PCa management
**NCT00699751** [36]	Interventional: Clinical Trial (Randomized)	• OS significantly improved	• GIT disorders, blood and lymphatic system disorders, nasopharyngitis, urinary tract infections, etc.	• Phase III trial using Radium-223 dichloride • 50k Bq/kg intravenously of body weight intravenously 4 cycles • Comparison with placebo
**NCT03511664 VISION TRIAL**	Interventional: Clinical Trial (Randomized)	• OS & rPFS	• No data yet posted	• ^177^Lu-617 in addition to best SOC • Estimated completion date December 2021
**REGISTRY NUMBER 12615000912583** **LUPSMA TRIAL**	Interventional: Clinical Trial (Randomized)	• OS • rPFS • Safety & tolerability	• Grade 1 dry mouth, grade ½ transient nausea, grade ½ fatigue and grade ¾ thrombocytopenia	• Phase 3 trial using ^177^Lu-PSMA-617 in progressive PSMA-positive mHRPC in combination with SOC • Comparison with SOC • No data yet as the study will be completed by December 2021
**NCT03454750** [37]	Interventional: Clinical Trial	• Disease control rate • Toxicity	• No data posted	• Radiometabolic therapy with ^177^Lu-PSMA-617 in HRPC (Lu-PSMA) • 7 GBq/cycle of ^177^Lu-PSMA-617 was safe & produced early biochemical & imaging responses • Dosimetry of salivary glands suggested that the co- administration of polyglutamate tablets may reduce salivary gland uptake
**NCT03403595**	Interventional: Clinical Trial	• Standardized uptake value of 177Lu-EB-PSMA-617 in normal organs & mHRPC	• Grade1/2 leucocyte reduction	• Phase I; ^177^Lu-EB-PSMA-617 in patients with mHRPC • The study was completed in December 2018
**NCT03828838**	Interventional: Clinical Trial	• Cancer Dose delivered to tumor and organs at risk	• No data posted	• Phase I/II; ^177^Lu-PSMA-617 in low-volume mHRPC • The study was completed in November 2019
**NCT03042468**	Interventional: Clinical Trial	• DLT • Recommended phase 2 dose	• Data not yet available	• Phase I Dose-escalation study of fractionated ^177^Lu-PSMA-617 for progressive mHRPC • Estimated completion date September 2022
**NCT03490838**	Interventional: Clinical Trial (Non-Randomised)	• DLT • PSA50 response rate	• Data not yet posted	• Phase I/II; ^177^Lu-PSMA-R2 in patients with PSMA-PET positive mHRPC • Estimated completion date June 2022
**NCT03276572**	Interventional: Clinical Trial	• Safety • DLT	• Low Gr temporary fatigue, nausea and xerostomia	• Phase I trial of ^225^Ac−J591 in Patients with mHRPC • Estimated completion date July 2024
**NCT03939689** [38]	Interventional: Clinical Trial	• PSA response rate	• The second and third therapies were less effective and presented with more frequent and more intense side effects, especially hematologic toxicities and xerostomia.	• Phase II study of ^131^I-PSMA-1095 radiotherapy in combination with enzalutamide in mHRPC patients who are chemo-naive and progress on abiraterone significantly reduced the tumor burden with low side effects. • The study is still ongoing and will be completed in June 2024
**NCT03792841**	Interventional: Clinical Trial	• Safety • DLT	• Data not yet published	• Phase I/II • Safety, tolerability, pharmacokinetics, and efficacy of AMG 160 in subjects with mHRPC • Estimated completion date November 2024
**NCT04053062**	Interventional: Clinical Trial	• Incidence of toxicity	• Data not yet published	• Phase I/II • PSMA-CAR T in treating patients with refractory mHRPC • Estimated completion date December 2022
**NCT03089203** [39]	Interventional: Clinical Trial (Non-Randomised)	• Incidence of toxicity	• Data not yet published	• Phase I • PSMA-TGFβRDN CAR T Cells for HRPC • Increased proliferation of lymphocytes, enhanced cytokine secretion, resistance to exhaustion, long-term in vivo persistence & the induction of tumor eradication was observed. • The study is ongoing & will be completed in September 2021
**NCT03577028**	Interventional: Clinical Trial	• DLT	• Data not yet published	• Phase I • Study of HPN424 in patients with advanced PCa • The study has been completed in December 2020
**NCT03545165**	Interventional: Clinical Trial	• DLT • Cumulative maximum tolerated dose • PSA response	• Data not yet published (Reporting date is July 2021)	• Phase I/II • ^177^Lu-J591 and 177Lu-PSMA-617 combination for mHRPC • The study has been completed in July 2020
**NCT00859781**	Interventional: Clinical Trial (Randomised)	• Proportion free of radiographic metastasis	• Data not yet published	• Phase II • ^177^Lu radiolabeled monoclonal antibody HuJ591 (^177^Lu-J591) and ketoconazole in patients with PCa • Estimated completion date December 2022
**NCT03093428**	Interventional: Clinical Trial (Randomized)	• Immune response evaluation • OS • rPFS • Safety & tolerability	• Data not yet published	• Phase II • Radium-223 + pembrolizumab (PD1). Radium-223 will be administered intravenously every 4 weeks at a pre-determined dose • The study is still ongoing and will be completed in 2024
**NCT02814669**	Interventional: Clinical Trial (Randomized)	• DLT assessment • OR	• No data published	• Phase I • Radium-223 + atezolizumab (PD-L1) • 840 mg intravenously infusion on days 1 & 15 of each 28-day cycle • Study completed but no data
**NCT02463799**	Interventional: Clinical Trial (Randomized)	• Immune response evaluation	• No data yet	• Phase II; Radium-223 with or without sipuleucel-T immunotherapy mHRPC • 50 kbq injected intravenously over 1 min per kg body weight per SOC every 4 weeks at weeks 0, 4, 8, 12, 16, and 20 • 6 infusions of radium-223 with 3 infusions of sipuleucel-T starting after second dose of radium-223 • 3 infusions of sipuleucel-T alone • The study will be completed in May 2021
**NOT REGISTERED** [40]	Prospective (Endotherapy)	• PSA Decline and stage	• Xerostomia	• Larger cohort required • Demonstrated the clinical impact of 225Actium-PSMA in managing patients with advanced PCa. • OS & PFS not endpoint

**ADT:** Androgen Deprivation Therapies; **AS:** Androgen Suppression; **AR-V7:** Androgen Receptor Variant; **CT:** Combination Chemotherapy; **CTLA4:** Cytotoxic T lymphocyte antigen-4; **DLT:** Dose limiting toxicity; **FFS:** Failure-Free Survival; **GBq:** Giga Becquerels; **GIT: Gastrointestinal tract**; **HR:** Hazard Ratio; **LT**: Long Term; **Lu:** Lutetium; **MFS:** Metastases free survival; **mHSPC:** metastatic Hormone Sensitive Prostate Cancer; **mHRPC:** metastatic Hormone Resistant Prostate Cancer; **NCT:** Number A unique identification code; **OS:** Overall Survival; **N/A:** Not applicable; **PCWG**: Prostate Cancer Clinical Trials Working Group; **PD1:** Programmed Cell Death Protein 1; **PDL1:** Programmed Death Ligand 1; **PFS**: Progression Free Survival; **PSA**: Prostate Specific Antigen; **PSMA:** Prostate Specific Membrane Antigen; **QoL:** Quality of Life; **RT**: Radiotherapy; **rPFS:** Radiological Progressive Disease; **SOC:** Standard of Care. To date, sipuleucel-T is the only immunotherapeutic agent that is FDA-approved for the treatment of HRPC and offers improved OS. Sipuleucel-T is an autologous antigen-presenting cell (APC) immunotherapy that destroys cancer cells by driving cytotoxic T cells into them. This therapy is associated with minimal side effects and accepted safety profile. However, only patients with the lowest PSA levels and tumour burden benefit from sipuleucel-T [41,42]. Although phase III trials with sipuleucel-T in mHRPC demonstrated an OS of 4-months when compared to placebo, the lack of positive effects when using surrogate end points underpins the need to have objective methodological criteria to assess efficacy [43]. Other concerning drawbacks include prolonged increased levels of PSA regardless of treatment, technically demanding process of therapy administration and highly costs of the drug that limits access of therapy to only patients in the Unites States [44,45]. Thus more biological and immunological biomarkers should be targeted for the development of more reliable and universal diagnostic markers and treatment. The existing PCa standard treatment is mainly a one-size-fits-all approach that is limited by the heterogeneity of the histological pattern grading, i.e., Gleason score, illustrated in Figure 1. It is therefore critical that future treatment developments consider these differences to benefit all cancer patients. In this perspective review, we will describe the role of co-inhibitory ICs in the light of their respective contributions to PCa treatment and its progression. We will also discuss peptide receptor ligand therapy (PRLT) strategies that can be used in combination to directly augment immune checkpoint-targeted therapy by exposing and unleashing evaded cancer cells through endoradiotherapy.

## Abbreviation

**Abbreviation**	**Full Name**
^225^Ac	^225^Actinium
^213^Bi	^213^Bismuth
^68^Ga	^68^Gallium
^177^Lu	^177^Lutetium
^223^Ra	^223^Radium
α	Alpha
ADT	Androgen deprivation therapy
AH	Androgen hormone
AS	Androgen Suppression
AR-V7	Androgen Receptor Variant
B7-H3	B7 homolog 3
BR	Biochemical recurrence
B7-H4	B7 Homolog 4
CDK12	Cyclin dependent kinase 12
CRP	C-reactive protein
CTLA4	Cytotoxic T-lymphocyte antigen 4
CT	Combination Chemotherapy
CTLA4	Cytotoxic T lymphocyte antigen-4
DCs	Dendritic cells
DLT	Dose limiting toxicity
DRE	Digital rectal examination
FFS	Failure-Free Survival
GAL9	Galectin-9
GBq	Giga Becquerels
GIT	Gastrointestinal tract
HMGB1	High-mobility group box 1
HR	Hazard Ratio
ICs	Inhibitory immune checkpoints
IDO	Indoleamine 2,3-dioxygenase
IFN-γ	Interferon-gamma
LDH	Lactate dehydrogenase
LT	Long Term
MSCs	Mesenchymal stem cells
mHRPC	Metastatic hormone-refractory PCa
mHSPC	Metastatic hormone-sensitive PCa
MFS	Metastases free survival
NCT	Number A unique identification code
NK	Natural killer cells
OS	Overall Survival
QoL	Quality of Life
PCa	Prostate cancer
PCWG	Prostate Cancer Clinical Trials Working Group
PD1	Programmed Cell Death Protein 1
PDL1	Programmed Death Ligand 1
PFS	Progression Free Survival
PSA	Prostate Specific Antigen
PSMA	Prostate-specific membrane antigen
PET	Positron Emission Tomography
PS	Phosphatidylserine
PRLT	Peptide receptor ligand therapy
RPT	Radical prostatectomy
RFS	recurrence free-survival
rPFS	Radiological Progressive Disease
RT	Radiotherapy
SOC	Standard of Care
SRT	Salvage radiotherapy
SPECT	Single Photon Emission Computed Tomography
SHP1/2	Src homology 2 (SH2) domain containing phosphatases 1/2
TIM-3	T cell Immunoglobulin Domain and Mucin Domain 3
TIGIT	T cell immunoreceptor with Ig and ITIM domains
TNF-α	Tumour necrosis factor-alpha
Tregs	Regulatory T cells
VTCN1	V-set domain-containing T cell activation inhibitor 1
